# Ten-Year Experience of Chest Wall Reconstruction: Retrospective Review of a Titanium Plate MatrixRIB™ System

**DOI:** 10.3389/fsurg.2022.947193

**Published:** 2022-07-05

**Authors:** Teddy H. Y. Wong, Ivan C. H. Siu, Kareem K. N. Lo, Ethan Y. H. Tsang, Innes Y. P. Wan, Rainbow W. H. Lau, T. W. Chiu, Calvin S. H. Ng

**Affiliations:** ^1^ Division of Cardiothoracic Surgery, Department of Surgery, The Chinese University of Hong Kong, Prince of Wales Hospital, Hong Kong SAR, China; ^2^ Division of Plastic and Reconstructive Surgery, Department of Surgery, The Chinese University of Hong Kong, Prince of Wales Hospital, Hong Kong SAR, China

**Keywords:** chest wall tumor, chest wall reconstruction, titanium rib plates, titanium ribs, patch repair

## Abstract

Chest wall tumor resection can result in a large defect that can pose a challenge in reconstruction in restoring chest wall contour, maintaining respiratory mechanics, and improving cosmesis. Titanium plates were first introduced for treating a traumatic flail chest, which yielded promising results in restoring chest wall stability. Subsequently, the applications of titanium plates in chest wall reconstruction surgery were demonstrated in case reports and series. Our center has adopted this technique for a decade, and patients are actively followed up after operation. Here, we retrospectively analyze our 10-year experience of using titanium plates and other reconstruction approaches for chest wall reconstruction, in terms of clinical outcomes, complications, and reasons for reoperation to determine long-term safety and efficacy. Thirty-eight patients who underwent chest wall resection and reconstruction surgery were identified. Of these, 11 had titanium plate insertion, 11 had patch repair or flap reconstruction, and the remaining 16 had primary closure of defects. Chest wall reconstruction using titanium plate(s) and patch repair (with or without flap reconstruction) was associated with larger chest wall defects and more sternal resections than primary closure. Subgroup analysis also showed that reconstruction by the titanium plate technique was associated with larger chest wall defects than patch repair or flap reconstruction [286.80 cm^2^ vs. 140.91 cm^2^ (*p* = 0.083)]. There was no 30-day hospital mortality. Post-operative arrhythmia was more commonly seen following chest wall reconstruction compared with primary closure (*p* = 0.041). Furthermore, more wound infections were detected following the use of titanium plate reconstruction compared with the patch repair (with or without flap reconstruction) approach (*p* = 0.027). In conclusion, the titanium plate system is a safe, effective, and robust approach for chest wall reconstruction surgery, especially in tackling larger defect sizes.

## Introduction

Chest wall tumor is a rare thoracic disease entity, and chest wall resection with reconstruction can be a challenging operation, particularly for large defects. To achieve a negative resection margin while maintaining the respiratory mechanism and cosmesis, surgeons have developed various ways to reconstruct the chest wall.

Methyl methacrylate is one of the most adopted methods for chest wall reconstruction. It requires a sandwiched technique, which is constructed with two outer layers of polypropylene mesh and a methyl methacrylate inner layer. However, due to the rigidity of the methyl methacrylate, significant post-operative pain and atelectasis can occur (1). Moreover, impermeability may lead to a higher risk of seroma formation and associated local infection (2). Due to methyl methacrylate's shortcomings, other materials and methods for reconstruction have been explored, such as polytetrafluoroethylene (PTFE) and titanium plate. Titanium plates like MatrixRIB™ (DePuy Synthes, USA) first emerged for use for stabilization of flailed segment(s) from rib fractures (3). The strong but flexible titanium plate offers several advantages. Firstly, the curved profile of the plate acts as a continuity of ribs in a more physiological way and maintains the “bucket-handle” mechanism. Secondly, it is easier to handle intra-operatively as it is precontoured and requires only two to three screws to secure into place. Moreover, titanium is nonferromagnetic and, thus, will cause less image artifacts in certain follow-up imaging scans. Titanium plates in chest wall reconstruction have, therefore, gained popularity, especially for large chest wall defects.

However, there is limited evidence to support the long-term safety and efficacy of titanium plate usage in chest wall reconstruction. There are only a few case reports illustrating the feasibility of using this plate for chest wall reconstruction (4–6). To the best of our knowledge, this study is the first retrospective study that looks into the long-term outcomes of patients who received titanium plates (MatrixRIB™, DePuy Synthes, USA) for chest wall reconstruction and to compare it with cases with primary repair or patch repair. By reviewing our results, we aim to evaluate the safety and efficacy of the titanium plate system over an extended time period.

## METHOD

### Patient Selection

From 2011 to 2020, clinical outcomes of patients who underwent chest wall reconstruction in the Prince of Wales Hospital were reviewed retrospectively.

Patients undergoing chest wall repair or reconstruction at our institute can broadly be divided into three groups: (a) primary closure of the defect by opposing surrounding soft tissue, (b) prosthetic patch repair reconstruction using synthetic or biocompatible material followed by closure of surface by surrounding soft tissue or autologous flap reconstruction, and (c) MatrixRIB™ titanium plate reconstruction using our previously reported approach (7).

Our institute will consider using titanium plate reconstruction when three or more ribs require resection, especially when anterior portions are resected. Occasionally, titanium plate reconstruction may be used following resection of two ribs if significant rib length and if a part of the sternum has also been resected.

The records of these patients were analyzed for age, sex, smoking status, comorbidities (ischemic heart disease, diabetes mellitus, hypertension, and chronic obstructive pulmonary disease), pathology of the lesion, and type of operation.

### Outcome Measures

The main outcome measures following the procedure include 30-day mortality, post-operative complications, duration of drain placement, length of stay, reasons and rate of reoperation, and late complications related to reconstruction.

### Statistical Analysis

Patients are classified as the primary closure group and those who received chest wall reconstruction as the chest wall reconstruction group (including those repaired with prosthetic patch or autologous flap reconstruction and those who received MatrixRIB™ titanium plate implantation). Subgroup analysis is also carried out between patients who received prosthetic patch repair or autologous flap reconstruction and MatrixRIB™ titanium plate implantation.

Categorical variables were reported as counts and compared between groups with the aid of the *χ*^2^ test. Continuous variables were reported as means and compared using the Student’s *t*-test. A two-tailed *p*-value threshold of 0.05 was used. All analyses were performed by using IBM SPSS Statistics 26.0.

## Results

### Patient Demographics

**Figure 1:**
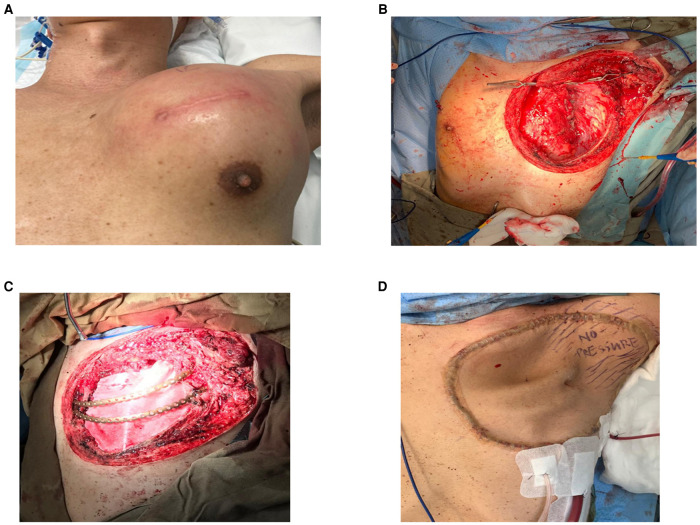
(**A**) Large left anterior chest wall poorly differentiated carcinoma involving the sternum, second to sixth ribs, and the left upper lobe of the lung. (**B**) After resection of tumor with the chest wall, hemisternectomy, left upper lobectomy. (**C**) Reconstructed with titanium ribs. (**D**) Following coverage with the anterior lateral thigh flap.

A total of 38 patients who underwent chest wall resection ± reconstruction were identified; 11 patients received implantation of MatrixRIB™ with or without additional prosthetic patch/autologous flap reconstruction (Figure 1; Supplementary Table S1), 11 patients received prosthetic patch (Gore® Dualmesh®, Gore Medical or Permacol™, Medtronic, USA) or autologous flap reconstruction only (Supplementary Table S2), and 16 patients received primary closure of defect (Supplementary Table S3). The patients’ age, sex, pre-operative work-up, and operative results are given in Tables 1–3 for the three groups, respectively. Of the 38 patients, 26 patients suffered from primary malignant/metastatic pathology, while 12 patients suffered from benign pathology. An analysis of the significant medical history of the patient group revealed that 10 patients were smokers (26%) and 6 were drinkers (15.7%), while 2 patients (5.2%) suffered from diabetes mellitus and 6 (15.7%) suffered from hypertension.

**Table 1:** Patient demographics—primary closure vs. chest wall reconstruction (both MatrixRIB™ implantation and prosthetic patch/autologous flap repair).

	Age (mean)	Sex	Smoking	Diabetes	Hypertension	Alcohol use
Primary closure (*n* = 16)	44.8 (range 5–83)	Male = 10(62.5%) Female = 6(37.5%)	7	0	4	4
Chest wall reconstruction (*n* = 22)	45.8 (range 2–81)	Male = 11(50%) Female = 11(50%)	3	2	2	2
*p*-value	*p* = 0.444	*p* = 0.886	*p* = 0.037	*p* = 0.215	*p* = 0.184	*p* = 0.154

**Table 2:** Operative variables—primary closure vs. chest wall reconstruction (both MatrixRIB™ implantation and prosthetic patch/autologous flap repair).

	Malignant pathology	Adjuvant therapy received with malignant pathology	Mean defect size (cm^2^)	Mean no. of ribs resected	No. of concomitant lung resection	No. of concomitant partial sternal resection	Mean operative time (minutes)
Primary closure (*n* = 16)	7(43.8%)	4(15.4%)	20 (SD 19.23)	1.4 (SD 1.12)	8	0	174.88 (SD 153.11)
Chest wall reconstruction (*n* = 22)	19(86.4%)	9(34.6%)	213.85 (SD 197.89)	2.59 (SD 1.40)	9	6	251.14 (SD 167.53)
*p*-value	*p* = 0.005	*p* = 0.307	*p* = 0.001	*p* = 0.01	*p* = 0.578	*p* = 0.023	*p* = 0.160

**Table 3:** Post-operative complications/outcomes—primary closure vs. chest wall reconstruction (both MatrixRIB™ implantation and prosthetic patch/autologous flap repair).

	Length of stay (days)	Duration of chest drain placement	Pneumonia	Arrhythmia	Wound infection	Reoperation required
Primary closure (*n* = 16)	Mean = 12.38 (SD 18.92)	Mean = 4.29 (SD 3.45)	1	0	0	3
Chest wall reconstruction (*n* = 22)	Mean = 19.77 (SD 15.85)	Mean = 8.26 (SD 9.69)	6	5	4	5
*p*-value	*p* = 0.737	*p* = 0.073	*p* = 0.099	*p* = 0.041	*p* = 0.071	*p* = 0.767

The primary closure group and chest wall reconstruction group were comparable in terms of age, gender, or significant comorbidities, except that more smokers were identified in the primary closure group (Table 1).

### Operative Variables

Several significant differences were noted between the primary closure and the chest wall reconstruction groups (Table 2). Notably, patients in the chest wall reconstruction group had significantly larger defects than those in the primary closure group (primary closure = 20 cm^2^ vs. chest wall reconstruction = 213.85 cm^2^, *t* = −3.89, *p* = 0.001). A larger number of ribs were resected (*t* = −2.741, *p* = 0.01) and more patients required partial sternal resection (*χ*^2 ^= 5.182, *p* = 0.023) in the chest wall reconstruction group compared with those in the primary closure group. More patients in the chest wall reconstruction group were also found to have suffered from malignant pathology compared with primary closure (*χ*^2^ = 7.785, *p* = 0.005). Significant differences were not demonstrated in other operative variables.

### Clinical Outcomes

No 30-day mortality occurred in any patient group. Post-operative complications and outcomes are given in Table 3. Patients with chest wall reconstruction had a higher incidence of arrhythmia than those with primary closure (*χ*^2 ^= 4.187, *p* = 0.041). There was a trend towards a longer duration of chest drain placement and a higher incidence of pneumonia and wound infection in the chest wall reconstruction group, but these did not reach statistical significance. No patient developed post-operative respiratory failure.

The post-operative follow-up duration was relatively long in those with chest wall reconstruction compared with primary closure but this was not significant (primary closure, mean = 1,202 days vs. chest wall reconstruction, mean = 17,405 days; *t* = 0.828, *p* = 0.093). In terms of long term complications (Table 3), among those 3 patients with primary closure requiring reoperation, 2 of them had recurrence of disease and proceeded for salvage operation, one patient with fibromatosis recurrence at right posterolateral costophrenic angle at 52 months from the index operation and the other patient had metastatic osteosarcoma at left lower lobe and required video-assisted thoracoscopic wedge resection at 47 months from the index operation; while the last patient suffered from MRSA (methicillin-resistant Staphylococcus aureus) pyogenic arthritis of the left sternoclavicular joint that need repeated drainage and delayed closure of wound after the index operation for clavicle and sternal resection. The cases of five patients requiring re-operation in the chest wall reconstruction group are discussed in the following section.

### Subgroup Analysis—MatrixRIB™ vs. Patch Repair/Flap

Patients in the chest wall reconstruction group were further analyzed with respect to their mode of reconstruction, namely, MatrixRIB™ implantation and prosthetic patch/autologous flap repair. The two groups show no significant difference in patient demographics (Table 4).

**Table 4:** Patient demographics—MatrixRIB™ implantation vs. prosthetic patch/autologous flap repair.

	Age (mean)	Sex	Smoking	Diabetes	Hypertension	Alcohol use
MatrixRib™ titanium plate implantation (*n* = 11)	40.63 (range 19–64)	Male = 7(63.6%) Female = 4(36.4%)	2	0	0	2
Prosthetic patch/autologous flap reconstruction (*n* = 11)	50.91 (range 2–81)	Male = 4(36.4%) Female = 7(63.6%)	1	2	2	0
*p*-value	*p* = 0.201	*p* = 0.22	*p* = 0.534	*p* = 0.138	*p* = 0.138	*p* = 0.138

For the operative variables, the MatrixRIB™ system was associated with the reconstruction of larger chest wall defects, while no other significant differences were observed (Table 5).

**Table 5:** Operative variables—MatrixRIB™ implantation vs. prosthetic patch/autologous flap repair.

	Malignant pathology	Mean defect size (cm^2^)	Mean no. of ribs resected	No. of concomitant lung resection	No. of concomitant partial sternal resection	Mean operative time (minutes)
MatrixRib™ titanium plate implantation (*n* = 11)	9(81.8%)	Mean = 286.8 (SD 257.06)	Mean = 2.73 (SD 1.49)	5	3	305.27 (SD 174.30)
Prosthetic patch/autologous flap reconstruction (*n* = 11)	10(90.9%)	Mean = 140.91 (SD 66.76)	Mean = 2.45 (SD 1.37)	4	3	197 (SD 148.69)
*p*-value	*p* = 0.534	*p* = 0.083	*p* = 0.826	*p* = 0.660	*p* = 1.000	*p* = 0.133

Post-operative complications and outcomes of chest wall reconstruction are outlined in Table 6. The length of hospital stay (*t* = 1.327, *p* = 0.199) and the duration of drain placement (*t* = 1.116, *p* = 0.280) were similar between the two reconstruction types, and despite the difference in the rate of re-operation, this was not statistically significant (*χ*^2^ = 2.329, *p* = 0.127). Wound infection rate was significantly higher in the MatrixRIB™ group (patch repair, *n* = 0 vs. MatrixRIB™ group, *n* = 4, *χ*^2 ^= 4.889, *p* = 0.027). None of the patients required removal of the prosthesis. Two patients had positive culture from pleural space and subcutaneous drain, which required ultrasound guided drainage of the collection and prolonged antibiotics with a 3-month interval. Two other patients had wound dehiscence within the post-operative first month, with both of them also undergoing concomitant flap reconstruction during the index operation. Out of these two, one had vacuum dressing for 2 weeks and then proceeded for debridement and partial skin graft, while the other had a debridement of wound 1 month after undergoing index operation and flap revision after 3 months. Interestingly, two patients with diabetes in our series who had prosthetic patch/autologous flap reconstruction did not suffer from any infectious complications from their wounds.

**Table 6:** Post-operative complications/outcomes—MatrixRIB™ implantation vs. prosthetic patch/autologous flap repair.

	Length of stay (days)	Duration of chest drain placement (days)	Pneumonia	Arrhythmia	Wound infection	Reoperation required
MatrixRib™ titanium plate implantation (*n* = 11)	Mean = 24.18 (SD 19.84)	Mean = 10.36 (SD 11.81)	3	1	4	4
Prosthetic patch/autologous flap reconstruction (*n* = 11)	Mean = 15.36 (SD 9.60)	Mean = 5.38 (SD 5.07)	3	4	0	1
*p*-value	*p* = 0.199	*p* = 0.280	*p* = 1.000	*p* = 0.127	*p* = 0.027	*p* = 0.127

Only one patient in the prosthetic patch/autologous flap reconstruction group required reoperation in the form of salvage surgery for tumor recurrence. She suffered from metastatic ductal carcinoma of the breast in the right middle lobe of the lung and underwent video-assisted thoracoscopic wedge resection at 24 months from undergoing index operation.

In the MatrixRIB™ implantation group, four patients had re-operations. One patient had salvage surgery for tumor recurrence of the poorly differentiated carcinoma with metastasis to the right chest wall at 5 months after index operation. Another patient suffered from concomitant autologous flap failure that needed revision in the same admission. The remaining two patients were operated upon for plate removal. Out of these, one had prosthesis exposed at 11 months after the initial operation, and the plate was removed and covered with a DIEP (deep inferior epigastric perforators) flap and further by a distant-free ALT (anterolateral thigh) flap as the DIEP flap failed, while the other patient presented with acute chest pain after a direct blow in a soccer game 25 months after operation and found that the plate fractured transversely (8). The plate was removed after an elective surgery in the same month as the patient had persistent pain.

## Discussion

Titanium plate systems like MatrixRIB™ have demonstrated excellent effect in stabilizing traumatic flail chest and rib fractures (3). By using the experience of treating fractures, surgeons have used titanium plates to help restore chest wall integrity when substantial reconstruction is needed, with mostly case reports being published in the past (6, 9). Compared with other conventional repair methods, titanium plates can be more ergonomic and user-friendly. The design of a titanium plate may also help reduce the risk of prosthesis fracture (7). Our retrospective series serves as the first one to document and analyze the long-term outcomes of chest wall reconstruction with titanium plates and to compare the clinical outcomes with other forms of chest defect repair in our center.

With regard to the primary outcome, in some larger-scale studies, the in-hospital mortality following complex chest wall resection and reconstruction was reported to be up to 7% (10). However, in our last 10 years of experience, no in-hospital or early post-operative mortality occurred. This may be explained by the improvement in surgical and anesthetic techniques and perioperative management in the past decade. The use of titanium plates allows better reconstruction of the chest wall to mimic normal anatomy and physiology, which could reduce the risk of respiratory failure and handling larger defects.

Although the titanium plate group had a higher risk of infection, which can be explained by the inherent risks of undergoing a more prolonged complex procedure involving prosthetic foreign body implantation, none of the infected cases required any operative intervention. Our practice of administering prophylactic antibiotic to cover the skin flora pre-operatively and for a few days post-operatively may be helpful in avoiding implant infections. Furthermore, we found that implant infection does not seem to be a long-term complication during our prolonged post-operative follow-up of these patients. We encountered a case of prosthesis fracture, which, in general, is a rare complication in the literature. The fracture was a sequela of physical impact during sports activity and a delayed fracture at 25 months after the index operation (8). Fortunately, we also did not encounter any previously reported post-operative complication of screw and plate loosening. Our practice of placing three screws into each plate end if possible, or placing only two screws into the end hole and a third one from the last hole, in order to provide more stability, may have helped avoid this complication.

Other disadvantages of titanium ribs are that they are permanent and rigid and can restrict growth, which is particularly a matter of concern in the pediatric patient population. Fortunately, for our youngest 2-year-old patient, the resected area was not so large to require a rigid reconstruction; therefore, a patch/autologous flap repair was sufficient. The data for chest wall reconstruction for pediatric patients are scanty, and the modalities for exactly how best to repair and reconstruct are still debated. In a setting where a rigid reconstruction is required in pediatric patients, special bars made of polylactic acid (BioBridge™, ACUTE Innovations, Hillsboro, OR, USA) can be implanted to provide immediate post-operative rigidity, which then gets absorbed after several months, limiting the disadvantages of a permanent implant.

There are a number of limitations in this study. Firstly, the retrospective nature of the study means the patient background and resections requiring reconstruction are heterogeneous. Furthermore, the decision with regard to the type of repair and reconstruction is dependent on the experience of the surgeon. Lesions that are large or malignant will result in larger chest wall defects to achieve adequate resection and negative margins. There will be a need for more complex reconstructions as primary repair provides inadequate coverage and support for tissue defects. Secondly, the study has a relatively small sample size and is exploratory in nature. There is no similar study previously to establish a benchmark for comparing the clinical outcome, especially concerning long-term post-operative complications with the use of MatrixRIB. However, the electronic patient database system of our region does allow a complete follow-up on all patients, documenting every clinical entry and detail. Thus, we are confident that our study has captured every clinical event and follow-up on patient status during their 10-year post-operative period. Finally, as the study was retrospective in nature and many patients were young, few underwent pre-operative or post-operative respiratory function tests, which could provide a more complete picture of the potential benefits of a more complex chest wall reconstruction. Therefore, future prospective studies are warranted, which can include measures of patient satisfaction, quality of life, and pre-operative and post-operative pulmonary functions using different reconstruction approaches.

## Conclusion

The titanium plate MatrixRIB™ System is a safe and effective approach for chest wall reconstruction surgery, particularly in tackling larger chest wall defects. Long-term complications arising from the use of titanium plates for chest wall reconstruction are few and manageable.

## Data Availability

The original contributions presented in the study are included in the article/**Supplementary Material**, and further inquiries can be directed to the corresponding author/s.
