# Supplementary material for: Ten-Year Experience of Chest Wall Reconstruction: Retrospective Review of a Titanium Plate MatrixRIB™ System
Source: Front Surg. 2022 Jul 5;9:947193. doi: 10.3389/fsurg.2022.947193 (PMC9294311; doi:10.3389/fsurg.2022.947193)
Supplement: Supplementary file 1 [file Table_1_v1.docx]

Suppl Table 1: Patients with implantation of MatrixRIB^TM^ with or without additional prosthetic patch/autologous flap reconstruction

Suppl Table 1: Patients with implantation of MatrixRIB^TM^ with or without additional prosthetic patch/autologous flap reconstruction

Suppl. Table 2: Patients with prosthetic patch or autologous flap reconstruction

IMAP=internal mammary artery perforator, ALT=anterolateral thigh, LD=latissimus dorsi , VRAM= vertical rectus abdominis myocutaneous

CT=computed tomography, MRI = magnetic resonance imaging, PETCT= positron emission tomography/computed tomography, USG=ultrasonography

Suppl. Table 3: Patients with primary closure of defect

CT=computed tomography, MRI = magnetic resonance imaging, PETCT= positron emission tomography/computed tomography, USG=ultrasonography
